# EARLY REHABILITATION IN TRAUMA PATIENTS: AN OBSERVATIONAL STUDY FROM TWO NORWEGIAN TRAUMA CENTERS

**DOI:** 10.2340/jrm.v58.45625

**Published:** 2026-06-30

**Authors:** Ingri GRIMNES OLSEN, Marianne WESNES, Hanne NÆSS, Eike WEHLING, Eirik VIKANE, Geir Arne SUNDE, Oddvar ULEBERG, Lars Gunnar JOHNSEN

**Affiliations:** 1 Department of Research and Development, The Norwegian Air Ambulance Foundation, Oslo; 2 Department of Circulation and Medical Imaging, Norwegian University of Science and Technology, Trondheim; 3 Regional Trauma Centre of Western Norway, Surgical Clinic, Haukeland University Hospital, Bergen; 4 Loddefjord Medical Centre, Bergen; 5 Department of Neurology, Haukeland University Hospital, Bergen; 6 Department of Clinical and Biological Psychology, University of Bergen, Bergen; 7 Department of Physical Medicine and Rehabilitation, Haukeland University Hospital, Bergen; 8 Department of Clinical Medicine, University of Bergen, Bergen; 9 Department of Anesthesia and Intensive Care, Haukeland University Hospital, Bergen; 10 Department of Emergency Medicine and Prehospital Services, St. Olav’s University Hospital, Trondheim; 11 Department of Neuromedicine and Movement Science, Norwegian University of Science and Technology (NTNU), Trondheim; 12 Department of Orthopaedic Surgery, St. Olav’s Hospital HF, Trondheim, Norway

**Keywords:** multiple trauma, rehabilitation, trauma centre, wounds and injuries

## Abstract

**Objective:**

To examine the provision of consultation by a specialist in physical and rehabilitation medicine, and timing of early rehabilitation services for severely injured trauma patients as recommended in the Norwegian Trauma Plan.

**Design:**

Observational cohort study.

**Subjects/Patients:**

Adults aged ≥ 18 years admitted with severe (New Injury Severity Score > 12) traumatic injuries.

**Methods:**

Observational cohort study with data from the Norwegian Trauma Registry and medical records. Outcomes included consultation with a physical and rehabilitation medicine specialist, timing of assessment, and involvement of health professions.

**Results:**

Among 317 patients, 10% received assessment by a physical and rehabilitation medicine specialist within 72 h. Early assessment occurred in 43% (*n* = 6) of patients with spinal cord injury, 12% (*n* = 8) with traumatic brain injury, and 7% (*n* = 17) with multitrauma. Overall, 31% (*n* = 99) of all patients were assessed by a physical and rehabilitation medicine specialist, including 93% (*n* = 13) of spinal cord injury patients, 57% (*n* = 39) with traumatic brain injury, and 19% (*n* = 43) with multitrauma. Physiotherapists assessed 72% of patients, while involvement from other rehabilitation professionals was limited.

**Conclusion:**

Despite recommendations, only one-third of patients were assessed by a physical and rehabilitation medicine specialist, and 10% within 72 h. Physiotherapy was common, but other rehabilitation input was limited. Patients with spinal cord injury received more comprehensive care than those with traumatic brain injury or multitrauma.

## LAY ABSTRACT

Severely injured patients often need rehabilitation during their hospital stay to support recovery and prevent complications. National Norwegian guidelines recommend a doctor specialized in physical and rehabilitation medicine should assess these patients within the first 72 h after injury. We examined how often this happens at two major trauma centres in Norway. Among 317 patients, only 1 in 10 received an early rehabilitation assessment, and only 1 in 3 saw a specialist in physical and rehabilitation medicine during their stay. Most patients were seen by a physiotherapist, but involvement from other rehabilitation professionals, such as occupational therapists, psychologists, and social workers, was limited. Patients with spinal cord injuries received the most comprehensive follow-up, while those with traumatic brain injuries or multiple injuries received far less. The results show that early rehabilitation is not provided consistently, and many patients may not receive the services they need for the best possible recovery.

Traumatic injury remains a major global public health challenge and is a leading cause of death and disability among persons aged under 45 years globally (1–4). Survivors frequently experience long-term functional impairments, even those with mild or no physical injuries, necessitating complex rehabilitation efforts (5–7). Although the importance of early rehabilitation in trauma care is increasingly recognized, standardized approaches extending beyond the first 24 h of treatment are still lacking (7–9). Understanding trauma survivors’ quality of life is essential for evaluating and improving the quality of care provided in the acute and post-acute phases (10).

Despite growing evidence supporting rehabilitation as an integral component of trauma management, it continues to receive limited attention in both clinical practice and organizational planning (11, 12). Several studies highlight the benefits of initiating multidisciplinary rehabilitation at the time of hospital admission (12–15). Early, coordinated intervention has been shown to reduce complications, promote physical recovery, and enhance health-related quality of life (HRQoL) (16). Optimal rehabilitation delivery ideally requires a multidisciplinary team, including a specialist in physical and rehabilitation medicine (PRM), physiotherapist, occupational therapist, speech and language therapist, clinical psychologist, nurse, social worker, and a rehabilitation coordinator (2, 9, 17).

International guidelines, including WHO’s “Guidelines for Essential Trauma Care” and National Institute for Health and Care Excellence (NICE) recommendations for rehabilitation after traumatic injury, underscore the importance of initiating early rehabilitation and ensuring its continuity throughout the care pathway (2, 9). The WHO’s 2017 report, “Rehabilitation 2030 – A call for action”, further highlights the growing global need for rehabilitation services as survival rates increase and people are becoming older (18). Evidence indicates that early rehabilitation, combined with seamless transitions across services, can reduce complications, accelerate recovery, and improve functional outcomes, particularly in patients with traumatic brain injury (TBI) and spinal cord injury (SCI) (13, 19, 20). Early rehabilitation has also been associated with reduced intensive care unit (ICU) and hospital lengths of stay and has demonstrated cost-effectiveness in TBI populations (13, 21, 22). However, there remains a notable lack of research examining early rehabilitation for patients with multiple injuries beyond TBI and SCI cohorts (13, 23).

The Norwegian National Trauma Plan (NTP) strongly recommends early rehabilitation for severely injured trauma patients, including consultation by a specialist in PRM within 72 h after admission, and direct transfer to rehabilitation units when indicated (8). Schäfer et al. (24) found low adherence to acute rehabilitation guidelines regarding early assessment by a PRM specialist and direct transfer from acute care to specialized rehabilitation centres. In addition, Moksnes et al. (12) revealed that 20% of trauma patients had unmet needs for subacute inpatient rehabilitation and 60% for community-based services within the first 6 months after moderate-to-severe traumatic injuries.

The aim of this study was to evaluate the provision of early PRM specialist consultation and early multidisciplinary rehabilitation at 2 Norwegian trauma centres, and to evaluate whether there are any differences between different type of injuries.

## METHODS

### Design and setting

An observational cohort study was conducted at 2 regional trauma centres in Norway, Haukeland University Hospital (HUS) in the Western region and St. Olav’s University Hospital (SOHO) located in the Central region of Norway. Together, these centres admit approximately 1,100 trauma patients annually, of whom about 1 in 4 are classified as severely injured, defined by an Injury Severity Score (ISS) > 15, a threshold associated with a high likelihood of long-lasting consequences (8, 25). The study adhered to the “Strengthening the Reporting of Observational Studies in Epidemiology” (STROBE) guidelines cohort studies (Fig. S1) (26). Primary outcomes were the prevalence of patients assessed by a PRM within 72 h and overall. Secondary outcomes were the prevalence and timing of involvement of patients assessed by other healthcare personnel (i.e., physiotherapy, occupational therapy, social worker, clinical nutritionist, speech therapist, psychologist).

At the time of the study, standardized care pathways existed for early specialized rehabilitation of patients with severe TBI (GCS < 9) and SCI, but no corresponding pathway had been established for multitrauma patients (19–21). At HUS, a specialist in PRM held a 40% position, working 2 days per week (excluding weekends and holidays). The PRM specialist followed patients from the ICU through the remainder of their hospital stay and occasionally in an outpatient clinic at the regional trauma centre. The responsibilities included clinical evaluation, initiation of early rehabilitation, and referrals to relevant healthcare professions such as physiotherapists, occupational therapists, speech therapists, and social workers. The PRM specialist also advised on the appropriate level of post-acute rehabilitation and discussed return-to-work or education with patients. At SOHO, patients routinely received physiotherapy, beginning in the ICU and continuing on the ward; however, treatment did not follow a multidisciplinary rehabilitation treatment model as recommended in the NTP (8). A specialist in PRM was involved only occasionally, typically for patients with central nervous system injuries such as TBI and SCI.

The study was performed in accordance with the Helsinki Declaration for Medical Research Involving Human Subjects. The Regional Committees for Medical and Health Research Ethics (REC) of Central Norway waived the need for patient consent (reference number 2019/798). The study was also approved by the Norwegian Centre for Research Data (reference number 626639). All data handling was performed according to General Data Protection Regulation and institutional approvals.

### Participants

The study included all patients aged ≥ 18 years admitted to HUS and SOHO and registered in the National Trauma Registry (NTR) between 1 January 2017 and 31 December 2017 with a New Injury Severity Score (NISS) > 12. Exclusion criteria were age < 18 years, death prior to hospital discharge, residence outside the Western or Central health regions, or transfers from other health regions.

A NISS > 12 was chosen because the commonly used ISS > 15 cutoff excludes some patients who nonetheless require long-term follow-up and rehabilitation (27). Both ISS and NISS are derived from the Abbreviated Injury Scale (AIS) methodology, which assigns a severity code and body region to each injury (28). ISS is calculated as the sum of squares of the 3 most severe injuries occurring in different body regions and ranges from 0 (minor) to 75 (worst outcome) (28). NISS uses the same formula but includes the 3 most severe injuries regardless of body region, improving accuracy, particularly in cases such as penetrating trauma and isolated head injury (27, 29). Both scores categorize injuries from ISS 0–8 (minor injury), ISS 9–15 (moderate injury), and ISS > 15 (severe injury/major trauma) (28). Patients were categorized according to their dominant injury into one of 3 groups: spinal cord injury (SCI), traumatic brain injury (TBI), or multitrauma(/other).

### Data collection

Demographic (age and gender) and clinical variables were extracted from the NTR and electronic patient records (EPJ) and transferred to a secure web-based case report form (WebCRF), forming the study database. Clinical variables included hospital admission (HUS/SOHO), type of injury (SCI, TBI or multitrauma), injury severity (ISS/NISS), assessment by a PRM specialist (yes/no), assessment within 72 h post-injury (yes/no), involvement of relevant health professionals (yes/no), and timing of involvement (days), as well as in-hospital rehabilitation stay (yes/no). Demographic variables, injury type, and injury severity were obtained from the NTR; all remaining variables were collected from the EPJ.

### Statistical analysis

Descriptive characteristics are reported as numbers and percentages for categorical variables. Continuous variables are presented as means and standard deviations (SD) or medians and interquartile range (IQR). Group differences in categorical variables were evaluated using Pearson’s χ^2^ test (two-sided). Fisher’s exact test was applied for sparse data, and the Mann–Whitney *U* test was used for non-normally distributed continuous variables. For all analysis a *p*-value < 0.05 was interpreted as statistically significant. Data analysis was performed using SPSS statistical software (released 2024. SPSS Statistics for Windows, Version 29, IBM Corp, Armonk, NY, USA).

## RESULTS

### Patient characteristics

A total of 317 severely injured patients were included in the study, with 159 treated at HUS and 158 at SOHO. The median age was 54 years (range 18–94) and 29% were women. Most patients had multitrauma (73%), followed by TBI (22%), and SCI (4%). At HUS, an additional 4 patients presented with major limb amputations (*n* = 2) or burn injury (*n* = 2). The overall mean ISS and NISS were 16 and 22, respectively. Patient characteristics for both centres are presented in Table I.

**Table I:** Patient characteristics of included patients for both centers

Factor	All *N* = 317	HUS *n* = 159	SOHO *n* = 158	*p*-value
Age (years)				0.340
Median (IQR)	54 (31)	54 (30)	54 (30)	
Mean (SD)	53 (19)	52 (19)	54 (19)	
Gender, *n* (%)				0.283
Female	93 (29)	42 (26)	51 (32)	
Male	226 (71)	117 (74)	109 (68)	
Type of injury, *n* (%)				
Head injury	68 (22)	31 (20)	37 (23)	0.395
Spinal	14 (4)	5 (3)	9 (6)	0.269
Multi	231 (73)	119 (75)	112 (71)	0.428
Amputation	2 (0.6)	2 (1)	N/A	0.498
Burn	2 (0.6)	2 (1)	N/A	0.498
Injury severity, median (IQR)				
ISS	14 (11)	14 (12)	14 (9)	0.334
NISS	17 (13)	17 (15)	17 (9)	0.229

HUS: Haukeland University Hospital; IQR: interquartile range; ISS: Injury Severity Score; *N*: total sample size; *n*: subgroup size; NISS: New Injury Severity Score; *p*-value: *p* < 0.05 considered statistically significant) SD: standard deviation; SOHO: St. Olav’s University Hospital.

### Assessment by PRM specialist

Of the 317 patients, 10% (*n* = 31) were assessed by a PRM specialist within the recommended 72 h. Among specific injury groups, 43% (*n* = 6) of patients with SCI, 12% (*n* = 8) of patients with TBI, and 7% (*n* = 17) of multitrauma patients received PRM specialist assessment within 72 h. Overall, 31% (*n* = 99) of all patients were assessed by a PRM specialist. The corresponding proportions were 93% (*n* = 13) for patients with SCI, 57% (*n* = 39) for those with TBI, and 19% (*n* = 43) for multitrauma patients. Sixty-three patients (20%) also received an in-hospital rehabilitation stay. Table II presents an overview of rehabilitation interventions. Table III presents how these findings were distributed between HUS and St. Olav’s.

**Table II:** Overview of rehabilitation interventions grouped by hospital

Factor	All *N* = 317	HUS *n* = 159	SOHO *n* = 158	*p*-value
Assessment by specialist in physical and rehabilitation medicine, *n* (%)	99 (31)	63 (40)	36 (23)	0.001*
Assessment within 72 h post-injury, *n* (%)	31 (10)	24 (15)	7 (4.5)	0.001*
In-hospital rehabilitation stays, *n* (%)	63 (20)	32 (20)	31 (20)	0.758

* Statistically significant; HUS: Haukeland University Hospital; *N*: total sample size; *n*: subgroup size; *p*-value: *p* < 0.05 considered statistically significant; SOHO: St. Olavs University Hospital.

**Table III:** Overview of rehabilitation interventions grouped by hospital and type of injury

Factor	Spinal cord injury		Traumatic brain injury		Multitrauma	
	HUS *n* = 5	SOHO *n* = 9	HUS *n* = 31	SOHO *n* = 37	HUS *n* = 119	SOHO *n* = 112
Assessment by specialist in physical and rehabilitation medicine, *n* (%)	5 (100)	8 (89)	18 (58)	21 (57)	36 (30)	7 (6)
Assessment within 72 h post-injury, *n* (%)	3 (60)	3 (33)	6 (19)	2 (5)	15 (13)	2 (2)
In-hospital rehabilitation stay, *n* (%)	5 (100)	7 (78)	14 (47)	19 (51)	11 (9)	5 (5)

HUS: Haukeland University Hospital; *n*: subgroup size; SOHO: St. Olav’s University Hospital.

### Assessment of other health professionals and time for involvement

Most patients were assessed by a physiotherapist (72%, *n* = 229), although one-third of severely injured patients did not receive such an assessment. Social workers were involved in 29% (*n* = 91), significantly more often at HUS (*p* < 0.0001). The distribution of these services between HUS and SOHO is presented in Table IV. Patients with SCI and TBI more frequently received multidisciplinary follow-up than multitrauma patients (Table V). Physiotherapists were the primary assessing profession across all groups. Two or more health professions were involved in 86% of all SCI cases and 63% of TBI patients. In contrast, among multitrauma patients, involvement from healthcare professionals other than physiotherapists remained limited.

**Table IV:** Proportion assessed by other health professionals grouped by hospital

Type	All *N* = 317	HUS *n* = 159	SOHO *n* = 158	*p*-value
Physiotherapist	229 (72)	120 (76)	109 (68)	0.145
Occupational therapist	86 (27)	50 (31)	36 (23)	0.072
Social worker	91 (29)	63 (40)	28 (18)	< 0.0001*
Clinical nutritionist	12 (4)	3 (2)	9 (6)	0.079
Speech therapist	33 (10)	13 (8)	20 (13)	0.205
Psychologist	44 (14)	23 (15)	21 (13)	0.728
Others^1^	28 (9)	7 (4)	21 (13)	< 0.0001*

* Statistically significant; HUS: Haukeland University Hospital; *N*: total sample size; *n*: subgroup size; *p*-value: *p* < 0.05 considered statistically significant; SOHO: St. Olav’s University Hospital;

1 Others: psychiatric nurse, pharmacist, orthopaedic engineer, educator, dentist.

**Table V:** Proportion of patients assessed by other health professionals grouped by type of injury.

Type	Spinal cord injury *n* = 14	Traumatic brain injury *n* = 68	Multitrauma *n* = 231
Physiotherapist	14 (100)	47 (69)	164 (71)
Occupational therapist	12 (86)	43 (63)	28 (12)
Social worker	12 (86)	34 (50)	41 (18)
Clinical nutritionist	3 (21)	5 (7)	3 (1)
Speech therapist	1 (7)	27 (40)	4 (2)
Psychologist	8 (57)	27 (40)	8 (4)
Others^1^	4 (29)	5 (7)	18 (8)

*n*: subgroup size;

1 Others: psychiatric nurse, pharmacist, orthopaedic engineer, educator, dentist.

Across both hospitals, median time from injury to involvement by physiotherapists was 2 days (IQR 2). Within the first 10 days, clinical nutritionists were the only additional health profession that consistently assessed patients (Table VI), although this applied to just 11 patients across both hospitals. There were significant differences between sites: physiotherapists, occupational therapists, and social workers became involved notably earlier at HUS compared with SOHO (Table VI). When stratified according to injury type, patients with spinal cord injuries were generally assessed earliest, followed by those with TBI, whereas multitrauma patients experienced the longest delays (Fig. S2). The wide variation within groups reflects substantial differences in how early rehabilitation services were initiated across the patient population.

**Table VI:** Time (days) from injury to involvement by other health professionals grouped by hospitals.

Type	Total Median (IQR)	HUS Median (IQR)	SOHO Median (IQR)	*p*-value
Physiotherapist	2 (4)	2 (2)	4 (4)	< 0.0001*
Occupational therapist	12 (18)	5 (8)	19 (17)	< 0.0001*
Social worker	9 (12)	6 (6)	28 (18)	< 0.0001*
Clinical nutritionist	26 (5)	26 (2)	26 (13)	0.954
Speech therapist	16 (19)	9 (5)	22 (22)	0.004*
Psychologist	21 (30)	19 (25)	29 (35)	0.748
Others^1^	6 (10)	6 (3)	5 (14)	0.763

* Statistically significant; HUS: Haukeland University Hospital; IQR: interquartile range; *p*-value: *p* < 0.05 considered statistically significant; SOHO: St. Olav’s University Hospital;

1 Others: psychiatric nurse, pharmacist, orthopaedic engineer, educator, dentist.

## DISCUSSION

This study evaluated the delivery and timing of early multidisciplinary rehabilitation services at 2 regional trauma centres in Norway, focusing on involvement by PRM specialists and other key health professionals, and examining differences between injury types. Overall, only 1 in 10 patients were seen within the recommended first 72 h by a PRM specialist and only 1 in 3 saw a PRM specialist at any point during their hospital stay. Although most patients received physiotherapy, involvement by other health professionals was limited. Patients with SCI were assessed more frequently than those with TBI or multitrauma and were more likely to receive early multidisciplinary rehabilitation.

A significantly higher proportion were assessed by a PRM specialist at HUS compared with SOHO, and more patients at HUS were evaluated within the first 72 h. This likely reflects the fact that HUS was the first regional trauma centre in Norway to employ a PRM specialist. Nevertheless, neither hospital met the NTP recommendations (8). Early PRM specialist involvement supports timely acute interventions and functional treatment, achieved through close collaboration with medical and surgical specialties. Several international guidelines emphasize the importance of early PRM consultation. The NICE guidelines strongly recommend that patients with an ISS > 8 should be assessed by a specialist in rehabilitation medicine within 72 h and receive a rehabilitation prescription (9). Similarly, the British Society of Physical and Rehabilitation Medicine has established standards for specialist rehabilitation within the trauma pathway and the American College of Surgeons advocates for rehabilitation to begin on the first day of hospitalization, with the aim of restoring pre-injury functional level (30, 31). Wagner et al. demonstrated that TBI patients who received PRM consultation within 48 h achieved significantly better Functional Independence Measure (FIM) scores and had significantly shorter acute hospital stays compared with those assessed later (32). Consistent with our findings, Schäfer et al. reported that only 18% of trauma patients received a PRM consultation within the first 72 h (24). PRM specialists may therefore play a crucial role in facilitating early multidisciplinary rehabilitation by initiating and coordinating the rehabilitation team and enabling timely transfer to specialized rehabilitation units when appropriate.

Establishing comprehensive trauma care pathways, from injury site to home, is recommended and should include early multidisciplinary rehabilitation as well as dedicated beds for multitrauma patients (8). Formalized collaboration between acute wards and rehabilitation units can facilitate faster transfers and reduce length of stay (LOS). Factors such as employment status, the presence of TBI or SCI, and longer ICU stays increase the likelihood of direct transfer to specialized rehabilitation, whereas patients with multitrauma are less likely to be transferred directly (32). At the time of our study, national care pathways existed for patients with SCI and TBI, but not for those with multitrauma (19, 20, 33, 34). In Norway, there are 3 regional specialized rehabilitation units for SCI; at HUS (Bergen), SOHO (Trondheim), and Sunnaas Hospital (Nesodden), and a few more regional specialized rehabilitation units for severe TBI; at HUS, SOHO, OUS (Oslo), Sunnaas Hospital, and UNN (Tromsø). Currently, there are no dedicated early rehabilitation units for multitrauma patients, even though this is strongly recommended (6). As a result, many of these patients may not receive the specialist rehabilitation that they require. Ideally, early multidisciplinary rehabilitation should be an integrated part of the comprehensive trauma care pathway in all severely injured trauma patients (35).

We observed substantial variation in both the timing and involvement of different healthcare professionals during the hospital stay. Patients with SCI received considerably more assessments, whereas those with multitrauma were less likely to be evaluated by multiple disciplines. Differences in the initiation of early rehabilitation and involvement of multidisciplinary teams at HUS and SOHO can partly be attributed to differences in organization and referral practices. At HUS, the PRM specialist collaborated closely with social workers, and all multitrauma patients with ISS > 12 were routinely referred to a social worker, who determined the need for further involvement based on the information in the medical records. SOHO had no comparable procedure, despite NTP recommending involvement of a social worker for all trauma patients < 75 years old (8). Several studies have demonstrated that integrating early rehabilitation into trauma care pathways reduces both mortality and LOS (13, 34, 36). Depending on the patient’s needs, early rehabilitation may range from a single therapeutic intervention to complex multidisciplinary approaches addressing both physical and cognitive impairments (2, 17, 37). However, previous studies indicate that only a minority of severely injured patients are assessed by multidisciplinary teams, consistent with our findings (12, 14, 32, 38). A persistent misconception remains that rehabilitation begins only after acute treatment, rather than being implemented in parallel with acute care as recommended by current guidelines (2, 37).

Although rehabilitation may be perceived as costly, inadequate rehabilitation after trauma can lead to even greater long-term societal and healthcare expenses. A recent study estimated the overall mean total healthcare cost per patient with moderate to severe injury at Norwegian Kroner (NOK) 846,877 (EURO 75,372) (39). An economic evaluation of rehabilitation after moderate and severe TBI found that rehabilitation generated an estimated value of NOK 540,000 (EURO 48,060) per patient (22). Maximizing long-term outcomes after trauma remains challenging, and randomized controlled trials investigating the impact of PRM follow-up in early trauma rehabilitation are needed, particularly to assess the cost-effectiveness of consultations by a PRM specialist and early multidisciplinary rehabilitation. Studies indicate that early rehabilitation and direct transfer to specialized rehabilitation units are cost-effective and associated with improved outcomes (13, 21). A recent systematic review was unable to draw firm conclusions regarding the benefits of early rehabilitation, underscoring the need for further research (23). More evidence is needed regarding early multidisciplinary rehabilitation for severely injured trauma patients beyond those with SCI and TBI. Such research is essential to support stratified care models and to guide clinicians and policymakers in further developing trauma rehabilitation services.

This study included all adults with NISS ≥ 12 treated in 2017 at 2 of Norway’s 4 regional trauma centres, covering the Central and Western health regions, and is therefore broadly representative for these regions but may not fully reflect national practice. Organizational differences between the centres, such as the presence of a dedicated PRM position at Haukeland but not at St. Olav’s, indicate that adherence to the Norwegian Trauma Plan and timing of early rehabilitation are sensitive to local resources and routines, and national estimates may therefore deviate from our figures. The findings are likely relevant for Scandinavian and other high-income trauma systems with comparable organization and access to specialist rehabilitation, but differences in staffing, formal care pathways, and transfer practices between countries, and especially between high- and lower-resource settings, limit direct extrapolation. Further multicentre and national studies are warranted to confirm these patterns and support context-specific implementation of early rehabilitation recommendations.

A possible limitation of this study is using retrospective data, as it is dependent on several registrars accurately recording data, which may affect the level and consistency of documentation. However, these recordings were performed by registered nurses certified in injury scoring and recorded the data as part of data collection to the (NTR), a national quality registry covering all trauma hospitals in Norway (25). Also, misclassification of exposures or outcomes is possible, particularly in cases with insufficient documentation. Key strengths include the large number of participants and the ability to compare data from 2 of the 4 regional trauma centres in Norway. By examining the involvement of all healthcare professionals, our findings demonstrate that clinical focus remains strongly oriented toward physical recovery.

Despite strong national and international recommendations for early consultation by a PRM specialist following severe injury, our study found that only one-third of the trauma patients were seen by a PRM specialist, of which only 10% within the first recommended 72 h. While most patients were seen by a physiotherapist, the involvement of other health professionals was limited. Considerable differences were observed between the two trauma centres and across injury types, with patients with SCI receiving more comprehensive rehabilitation than those with TBI or multitrauma.

## Supplementary Material


