# Supplementary material for: EARLY REHABILITATION IN TRAUMA PATIENTS: AN OBSERVATIONAL STUDY FROM TWO NORWEGIAN TRAUMA CENTERS
Source: J Rehabil Med. 2026 Jun 30;58:45625. doi: 10.2340/jrm.v58.45625 (PMC13329718; doi:10.2340/jrm.v58.45625)
Supplement: Supplementary file 1 [file JRM-58-45625-s1.pdf]

Fig. S1. STROBE statement for cohort studies.

STROBE Statement—Checklist of items that should be included in reports of ***cohort studies***

	Item No	Recommendation	Page No
**Title and abstract**	1	(*a*) Indicate the study's design with a commonly used term in the title or the abstract<br>(*b*) Provide in the abstract an informative and balanced summary of what was done and what was found	1 / 3
**Introduction**			
Background/rationale	2	Explain the scientific background and rationale for the investigation being reported	6
Objectives	3	State specific objectives, including any prespecified hypotheses	7
**Methods**			
Study design	4	Present key elements of study design early in the paper	7
Setting	5	Describe the setting, locations, and relevant dates, including periods of recruitment, exposure, follow-up, and data collection	7
Participants	6	(*a*) Give the eligibility criteria, and the sources and methods of selection of participants. Describe methods of follow-up<br>(*b*) For matched studies, give matching criteria and number of exposed and unexposed	8
Variables	7	Clearly define all outcomes, exposures, predictors, potential confounders, and effect modifiers. Give diagnostic criteria, if applicable	7 / 8
Data sources/ measurement	8*	For each variable of interest, give sources of data and details of methods of assessment (measurement). Describe comparability of assessment methods if there is more than one group	8
	9	Describe any efforts to address potential sources of bias	N/A
Study size	10	Explain how the study size was arrived at	N/A
Quantitative variables	11	Explain how quantitative variables were handled in the analyses. If applicable, describe which groupings were chosen and why	8 / 9
Statistical methods	12	(*a*) Describe all statistical methods, including those used to control for confounding<br>(*b*) Describe any methods used to examine subgroups and interactions<br>(*c*) Explain how missing data were addressed<br>(*d*) If applicable, explain how loss to follow-up was addressed<br>(*e*) Describe any sensitivity analyses	8 / 9
**Results**			
Participants	13*	(a) Report numbers of individuals at each stage of study—eg numbers potentially eligible, examined for eligibility, confirmed eligible, included in the study, completing follow-up, and analysed<br>(b) Give reasons for non-participation at each stage<br>(c) Consider use of a flow diagram	9
Descriptive data	14*	(a) Give characteristics of study participants (eg demographic, clinical, social) and information on exposures and potential confounders<br>(b) Indicate number of participants with missing data for each variable of interest<br>(c) Summarise follow-up time (eg, average and total amount)	9
Outcome data	15*	Report numbers of outcome events or summary measures over time	9

Fig. S2. Time to involvement by health professionals by injury type.

The boxplot represents the interquartile range (IQR), with the horizontal line indicating the median and variability outside the upper and lower quartiles. Whiskers extend to the lowest and highest values within 1.5 × IQR. Points outside this range are plotted as outliers. Patients with spinal cord injuries were generally seen earliest, followed by those with head injuries, whereas multitrauma patients experienced the longest delays. The wide variation within groups reflects substantial differences in how early rehabilitation services were initiated across the patient population.

Supplemental File 1. Time to involvement by health professionals by injury type

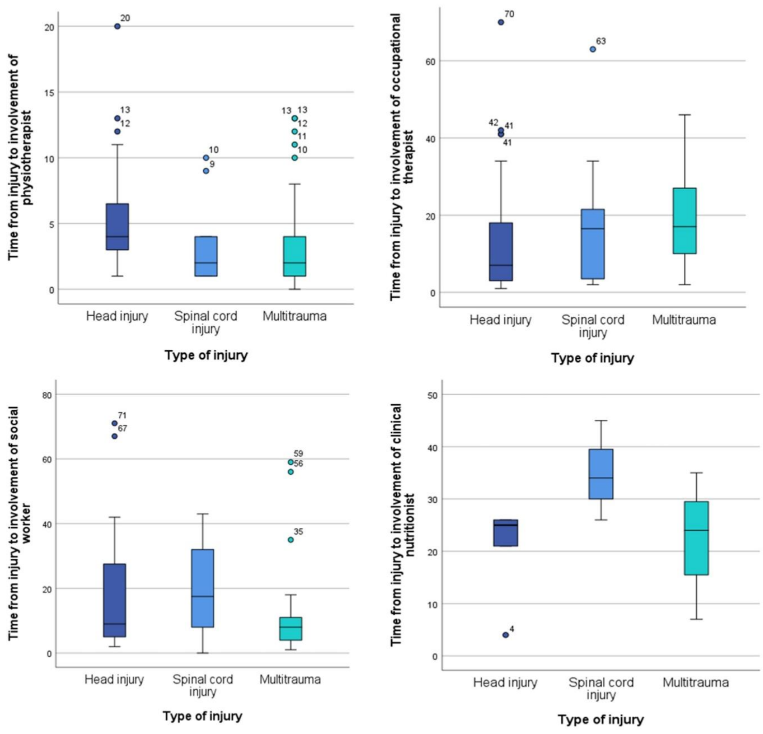

Åpen
